# *In Vitro* Detection of Caries Around Amalgam Restorations Using Four Different Modalities

**DOI:** 10.2174/1874210601711010609

**Published:** 2017-12-12

**Authors:** Tamara E. Abrams, Stephen H. Abrams, Koneswaran S. Sivagurunathan, Josh D. Silvertown, Warren M.P. Hellen, Gary I. Elman, Bennett T. Amaechi

**Affiliations:** 1 Quantum Dental Technologies Inc, Toronto, Ontario, Canada; 2 Cliffcrest Dental Office, Scarborough, Ontario, Canada; 3 University of Texas Health Science Center, San Antonio, Texas, USA

**Keywords:** Laser fluorescence, Canary System (CS), LED fluorescence (Spectra), Visual inspection (ICDAS II), Margin of the restoration (MOR), Amalgam restorations

## Abstract

**Objective:**

The aim of this study was to evaluate the ability of PTR-LUM (The Canary System, CS), laser fluorescence (DIAGNOdent, DD), LED fluorescence (Spectra), and visual inspection (ICDAS II) to detect natural decay around bonded amalgam restorations *in vitro*.

**Methods:**

Seventeen extracted human molars and premolars, consisting of visually healthy (n=5) and natural cavitated (n=12) teeth were selected. For the carious teeth, caries was removed leaving some decayed tissue on the floor and or wall of the preparation. For sound teeth, 3 mm. deep cavity preparations were made and teeth were restored with bonded-amalgam restorations. Thirty-six sites (13 sound sites; 23 carious sites) were selected. CS and DD scans were performed in triplicate at 2, 1.5, 0.5, and 0 mm away from the margin of the restoration (MOR). Spectra images were captured for the entire surface, and dentists blinded to the samples provided ICDAS II scoring.

**Results:**

Canary Numbers (Mean±SE) for healthy and carious sites at 2, 1.5, 0.5, and 0 mm from the MOR ranged from 12.9±0.9 to 15.4±0.9 and 56.1±4.0 to 56.3±2.0, respectively. DD peak values for healthy and carious sites ranged from 4.7±0.5 to 13.5±2.99, and 16.7±3.7 to 24.5±4.4, respectively. For CS and DD, sensitivity/specificity for sites at 2.0, 1.5, 0.5, 0 mm ranged from 0.95-1.0/0.85-1.0, and 0.45-0.74/0.54-1.0, respectively. For ICDAS II, sensitivity and specificity were 1.0 and 0.17, respectively. For Spectra, data and images were inconclusive due to signal intereference from the amalgam restoration.

**Conclusions:**

Using this *in-vitro* model, CS and DD were able to differentiate between sound and carious tissue at the MOR, but larger variation, less reliability, and poorer accuracy was observed for DD. Therefore, CS has the potential to detect secondary caries around amalgam restorations more accurately than the other investigated modalities.

## 1 INTRODUCTION

Secondary caries is one of the major reasons for the replacement of amalgam restorations [1]. Caries detection around the margins of restorations including amalgams is a major challenge in clinical practice. Typically older amalgam restorations may cause some marginal staining but visually the margins may appear intact and sound. The detection of secondary caries in its early stages is not easy [2], especially with current detection methods including radiography, explorer, fluorescence based devices and visual examination [3]. Discoloration next to the restoration or ditched amalgam margins is not necessarily predictive of secondary caries [4-6]. But, visual or visual-tactile examination often in combination with bitewing radiographs, are still the most common methods for caries detection in clinical practice [7].

The International Caries Detection and Assessment System (ICDAS II) visual criteria were introduced to assist in caries detection [8]. The surface characteristics from secondary caries are considered similar to primary caries so the criteria used for ICDAS II ranking of primary caries can also be applied to caries around restorations (CARS) [9, 10]. Research has shown that the ICDAS presents good reproducibility and accuracy for *in vitro* and *in vivo* detection of primary caries lesions at different stages of the disease [10-12].

Caries detection methods, such as laser fluorescence (DIAGNOdent 2095 [LF], KaVo, Biberach, Germany) have been used as aids in the detection of demineralized dental tissue beneath restorations [7, 13]. In 2006, a laser fluorescence device (DIAGNOdent 2190 [LFpen], KaVo) was developed to assist the detection of occlusal and approximal caries. The LFpen is able to capture, analyze, and quantify the fluorescence emitted from bacterial porphyrins and other chromophores when the tooth is illuminated by a diode laser with a wavelength of 655 nm [14, 15]. **In-vitro** studies have shown that the LF can detect caries at amalgam margins but stain and amalgam overhang do reduce the sensitivity [16-18].

The Spectra Caries Detection System using fluorescence technology light-emitting diodes (LED) projects high-energy light onto the tooth surface causing cariogenic bacteria to fluoresce red and healthy enamel green [19, 20] The device emits a light with a 400-nm wavelength and filters the fluorescence emitted by the tissue. Specific software then quantifies the fluorescence on a numerical scale from 0 to 5 [21]. This device also captures the fluorescence from bacterial porphyrins [20, 22, 23]. Some studies have demonstrated the ability of SPECTRA to detect caries on occlusal surfaces [24-27] but the detection around restoration margins or beneath sealants may be more challenging [28, 29].

The Canary System uses energy conversion technology (PTR-LUM) to image and examine the tooth. Pulses of laser light are aimed at the tooth, and the light is then converted into heat (Photothermal Radiometry or PTR) and light (luminescence or LUM), which are emitted from the tooth surface between pulses. These pulses of laser light enable the clinician to examine lesions up to 5 mm, below the surface [30, 31]. Caries modify the thermal properties (PTR) and luminescence (LUM) of healthy teeth. As a lesion grows, there is a corresponding change in the signal. In effect, the heat confined to the region with crystalline disintegration (dental caries) increases the PTR and decreases the LUM. As remineralization progresses and enamel prisms start to reform their structure, the thermal and luminescence properties begin to revert towards those of healthy tooth structure [32-35].

This study explored the ability of various caries detection systems to detect secondary caries around and beneath the margins of amalgam restorations. The experimental model does mimic one possible clinical situation where margins are intact but secondary caries is developing beneath the restoration margin. This *in-vitro* model may provide the clinician and researcher with information on which caries detection systems can be used clinically.

## 2 MATERIALS AND METHODS

### 2.1 Study Design

Seventeen permanent extracted human teeth (molars and premolars) consisting of 5 visually sound and 12 teeth with natural cavitated lesions were selected. The teeth were cleaned to remove any surface stain or debris but the lesions were left undisturbed. Tooth samples were stored in distilled water before and after each examination or measurement to avoid dehydration using the protocol established in our earlier studies [30, 36, 37]. Each tooth sample in the study was removed from the vial, rinsed thoroughly with clean distilled water for 20 seconds, and air-dried for five seconds before visual examination or measurements were taken.

One dentist selected the smooth surface to be restored on both the visually sound and caries samples. A standard amalgam preparation was done using high speed hand piece bur to remove any hard tissue and a slow speed hand piece with round carbide bur to remove dentin and caries. On the sound samples the cavity preparation was at least 3 mm in depth. On the samples with caries, the caries was removed, except on one wall. On that wall, the caries and demineralized enamel was removed from the preparation margin but caries remained at least 1 mm below the tooth surface. All measurements were done using a standard periodontal probe (Williams Periodontal Probe PW6 Hu-Friedy Chicago Illinois).

Once the preparations were completed, the teeth were photographed on all surfaces and then the amalgam restoration was placed. Standard bonded amalgam technique was used. The cavity preparation was etched for 30 seconds using 37% phosphoric acid gel (Temrex Gel Etch). The teeth were rinsed with water for 30 seconds. to ensure that all the phosphoric acid gel was removed. They were air dried for 30 seconds. Bond1 Primer / Adhesive (Pentron Clinical Technologies) was used to bond each amalgam. Equal parts of Primer A was mixed with Primer B and applied to the interior of the preparation. A dental curing light (Demi-Ultra LED Curing Light Kerr Orange County California) was shone on the preparation for 20 seconds to cure this layer. Then equal parts of Bond-It Resin - Light Cure and Dual-Cure Activator (Pentron Clinical Technologies) were mixed and applied to the interior of the preparation. Amalgam (Dispersalloy Dentsply Sirona) was mixed for 20 seconds in an amalgamator, according to manufacturer’s instructions and then placed into the preparation. An amalgam condenser, compacted the amalgam and more amalgam was added until the preparation was completely filled. The margins were cleaned of amalgam and any other material. The restoration was left to set and then placed back into a vial of distilled water.

Photographs were taken of all the surfaces of all the teeth with the restorations in place. On each photograph a section of the amalgam margin was selected for examination. On samples with caries beneath the amalgam, a section of the margin with caries was selected. The Canary System and DIAGNODent measured point scans up to 2 mm away from the amalgam margin. This was the maximum distance one could measure on the carious samples before moving on to the adjacent surface.

Another operator took DD and CS measurements at the centre of the amalgam, margin of the amalgam, 0.5 mm 1.5 mm and 2.0 mm away from the margin. Each measurement was done three times and all measurements were recorded. The means and standard deviation for each measurement were calculated. The measurement scales for the various systems are shown in Fig. (**1**).

### 2.2 ICDAS II Visual Examination

Two blinded dental clinicians, each trained and experienced in caries detection and diagnosis using ICDAS II visual scoring system, were given sample teeth and were asked to score each the tooth surface with the amalgam restoration independently. The ICDAS II criteria were: 0: Sound tooth surface; 1: First visual change in enamel; 2: Distinct visual change in enamel; 3: Localized enamel breakdown due to caries with no visible dentine or underlying shadow; 4: Underlying dark shadow from dentine; 5: Distinct cavity with visible dentine; 6: Extensive distinct cavity with visible dentine and more than half of the surface involved. All visual examinations were conducted under standard conditions in a dental operatory with dental operatory light and no visual aids. Where there was disagreement between the clinicians’ scores, surfaces with amalgam restorations were re-examined by both clinicians together and a consensus score reached. The consensus score was recorded.

### 2.3 Spectra Caries System Assessment

The Spectra Caries Detection Aid System (2010 Allpro Imaging Spectra Caries Detection Diagnostic System) captured an image of each tooth surface being assessed. The images were stored on a netbook using Spectra Imaging software. A 10-mm distance spacer and the Spectra handpiece disposable camera covers were used (AIR TECHNIQUES, Melville New York).

### 2.4 Diagnodent Assessment

DIAGNOdent Classic (KAVO model 2095, Biberach, Germany) was used in accordance with the manufacturer’s operating instructions, using probe “A” to allow for point measurements at various distances from the amalgam margin. For each tooth, the device was calibrated with a calibration disc and a zero baseline was established using a sound spot. Each tooth was air-dried for five seconds and the tip of the DIAGNOdent was placed perpendicular to the examination site. Three measurements were taken for each site and the mean peak value was calculated.

### 2.5 The Canary System Assessment

The CS was used in accordance with the manufacturer’s operating instructions to obtain readings from the tooth surface. The device was calibrated in accordance with the manufacturer’s calibration instructions. Each tooth was air-dried for five seconds and the cone of the disposable plastic tip on the handpiece was positioned over the examination site and a scan was taken. Three measurements were taken at each position.

### 2.6 Blinding of the Operators and Clinicians

A number of steps were taken to blind each of the operators and clinicians in this study. The samples for inclusion in the study were selected by one operator who also placed the restorations. A second operator did the examinations with CS, DD and SPECTRA. Two clinicians were then asked to rank these sites using ICDAS II criteria and then review their findings and come to an agreement on the ranking. Statistical analysis was done by third operator.

### 2.7 Statistical Analysis

Since the teeth had been pre-selected as sound and carious before examination with the various systems, they were divided into these two groups for analysis. Sensitivity and specificity analysis were performed on the data collected using The CS, SPECTRA, ICDAS II and DD. The ICDAS rankings were only done for the tooth surface under observation. The mean numbers for CS and DD were analyzed at the margin of the restoration, 0.5 mm, 1.5 mm and 2 mm away from the amalgam.

## 3 RESULTS

Two dental clinicians examining the margins of the amalgam restoration using ICDAS II rankings were only able to locate two carious margins. On these two margins the agreed ICDAS ranking was 3. All the other amalgam margins on both carious and healthy samples were ranked as ICDAS 0 (healthy). The sensitivity and specificity were 1.0 and 0.17 respectively. Visual ranking with ICDAS II was not an accurate method for detecting caries beneath amalgam restoration margins.

**Fig. (2):**
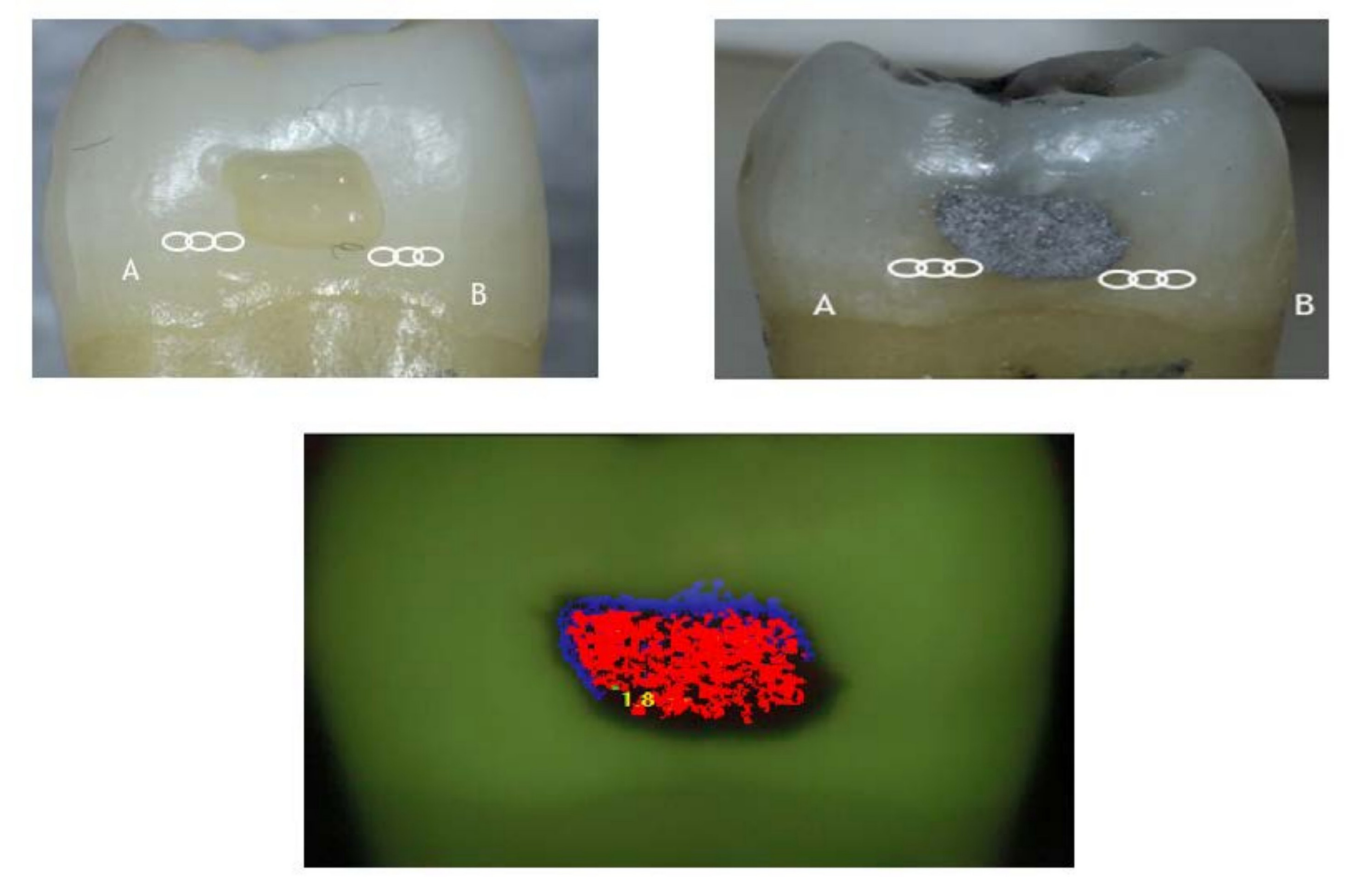
Representative sound tooth before (A) and after (B) amalgam placement. The circles indicate triplicate measurements taken with SPECTRA at MOR, 0.5, 1.5 & 2.0 mm away from amalgam margin.

**Fig. (3):**
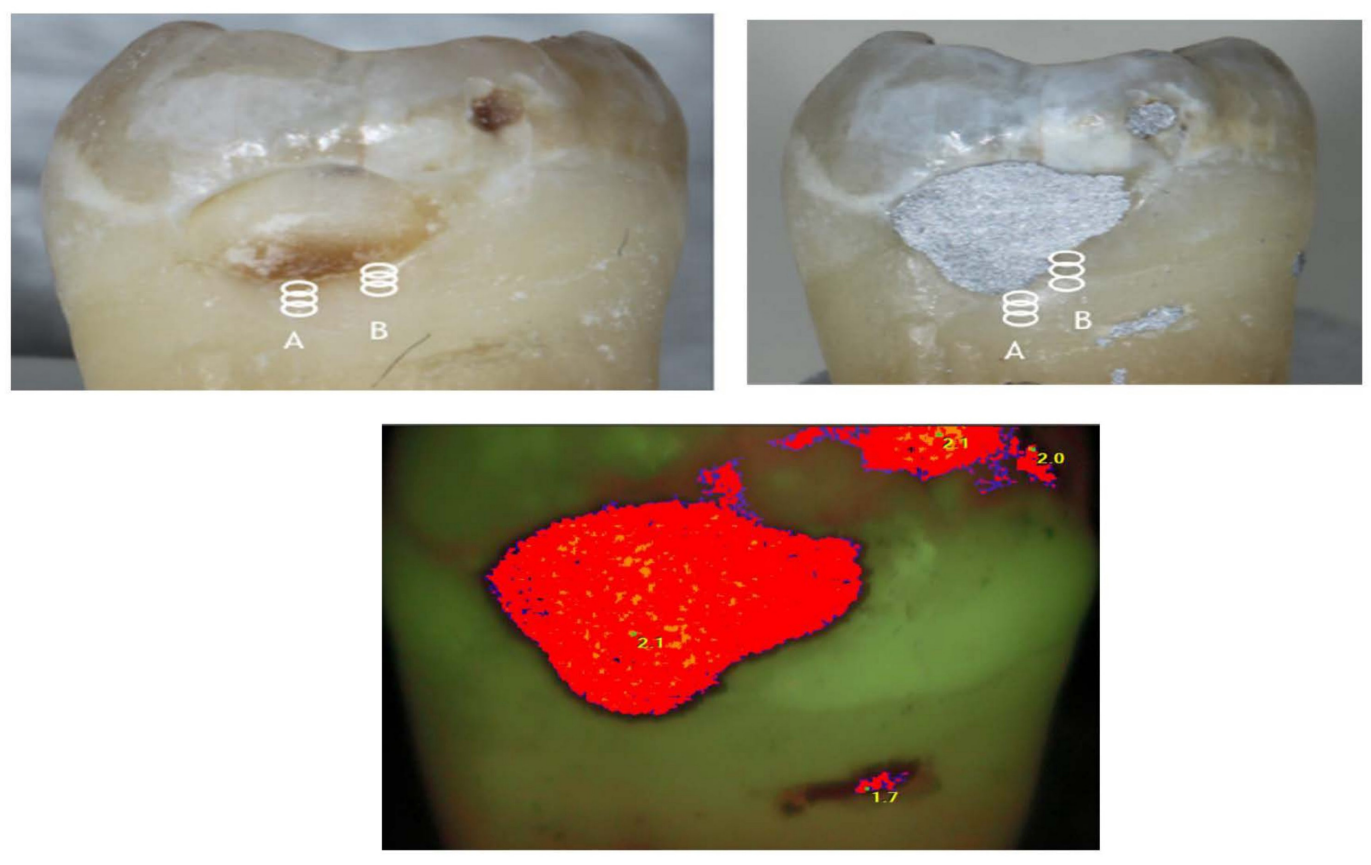
Representative carious tooth before (A) and after (B) amalgam placement. The circles indicate triplicate measurements taken with SPECTRA at MOR, 0.5, 1.5 & 2.0 mm away from amalgam margin.

SPECTRA images of the amalgam restoration were all red indicating deep enamel caries. Along the margins of all the amalgam restorations, there were very thin blue or black lines (Figs. **2** and **3**). The remaining tooth surface appeared green indicating sound enamel even if caries was present beneath the amalgam margin. SPECTRA measured surface fluorescence using a 405 nm wavelength of LED light. The amalgam had very high reflectivity so SPECTRA was not able to accurately image or measure fluorescence around the amalgam margin. The sensitivity and specificity at the amalgam margin were 1.0 and 0.0 respectively. At 2 mm away from the amalgam margin the sensitivity and specificity were 0.0 and 1.0 respectively.

Figs. (**4** and **5**) show the mean Canary Number and DIAGNODent readings on the centre of the amalgam restoration and at various distances from the margins of the restoration. The Canary system gave readings of 46.2 ± 8.4 and when measuring on the centre of the amalgam restoration on the healthy and carious samples. At the margin of the restoration, the CN reading from carious samples were 56.3 ± 9.4. On healthy samples at the margin of the restoration the CN dropped to 15.4± 3.1. The measurements at 0.5, 1.5 and 2.0 mm away from the amalgam margin on healthy samples the CN remained below 20 indicating no caries present. However, on teeth with caries beneath the amalgam margins the Canary Number measurements at 0.5, 1.5 and 2.0 mm away from the amalgam margin gave mean ranged between 56.1 and 58.9 indicating that there was caries beneath the margin or near to the margin wall underneath the surface enamel. The Canary Number did not drop significantly at 2 mm away from the amalgam margin on carious samples. Table **1** indicated that The Canary system had very high sensitivity and specificity rankings. For Canary System, sensitivity/specificity for sites at 2.0, 1.5, 0.5, and on the amalgam margin ranged from 0.95-1.0 / 0.85-1.0, respectively (Table **1**).

DIAGNODent gave readings of 12.1 ± 7.8 when taking measurements on the middle of the amalgam restoration for both sound and carious samples. At the margin of amalgams placed in healthy teeth the DIAGNODent reading was 13.5± 10.4 and dropped to 4.7± 1.8 at 2 mm away from the amalgam margin. On teeth with caries beneath the amalgam margin DIAGNODent readings at the margin were 24.5 ± 20.8 and dropped to 16.7 ± 16.5 at 2mm away from the amalgam margin. For DIAGNODent, sensitivity/specificity for sites at 2.0, 1.5, 0.5, and on the amalgam margin ranged from 0.74-0.52 / 0.54-1.0 (Table **1**).

Table **2** shows the results of Two Sample T-Tests for The Canary System and DIAGNODent readings on carious and healthy samples. In Two Sample T-Test, The Canary System and DIAGNODent readings taken at the center of the amalgam were tested with corresponding readings at the margin of restoration, 0.5mm, 1.5mm and 2 mm away for both healthy and carious samples. T-Test results on The Canary system showed population variance almost equal for healthy and carious samples at the margin of restoration as well as 0.5mm, 1.5mm and 2mm away (<0.05). T-Test results from Table **2** confirm with sensitivity/specificity results from Table **1** for The Canary System. Using The Canary System 0.5mm to 2.0 mm away from the margin of restoration is the best option for caries detection compared to scanning at the margin of the restoration.

T-Test on DIAGNODent showed population variance wasn’t equal for all results on healthy samples (>0.05), except at 2 mm distance from the amalgam margin. The DIAGNODent results on carious samples showed that at 1.5mm and 2.0 mm population variance wasn’t equal for (>0.05). T-Test on DIAGNODent (Table **2**) agree with sensitivity/specificity results (Table **1**) showing that relatively better sensitivity at margin than at 0.5 mm to 2.0 mm for carious samples but the specificity for DIAGNODent had lower variances at 2.0 mm for healthy samples. Conflicting result for DIAGNODent for carious and healthy samples is a potential concern when using DIAGNODent primarily to detect caries near the amalgam margins. In this study, newly made amalgam filling had polished surfaces with no biofilms, surface abrasions or stains on them. But in clinical situations with older amalgam restorations, the uncertainty of DIAGNODent measurements at the margin of the restoration will be even higher with biofilms, stains wear and bacteria on the amalgam impacting upon the DIAGNODent reading.

## 4 DISCUSSION

Detection of caries around and beneath the margins of amalgam restorations is one of the many challenges in clinical practice. In particular, the relevance of both marginal ditching and staining around amalgam restorations is unclear [4, 5]. In this study we examined the ability to detect caries around the visible margins of amalgam restorations *in vitro*. This situation would occur clinically on amalgam margins located on occlusal and smooth surfaces. Since the introduction of resin composite more than five decades ago, there has been a constant decline in the use of amalgam as restorative material [38], but one still needs to monitor and assess the marginal integrity of amalgam restorations for the presence of caries.

The development of lesions adjacent to existing clinical restorations is a multifactorial problem that is difficult to study due to human variability and the time required for identifiable lesion formation [39]. This experimental model does not exactly mirror a typical clinical situation. Clinically a restoration is placed into a cavity preparation that has sound caries free walls. This *in vitro* model was chosen in order to simulate a cavity wall lesion which would develop months or years after the placement of a restoration. The objective of the study was to see if various caries detection systems could detect caries beneath the visible intact margins of an amalgam restoration.

Visual or visual-tactile examinations (use of explorer), often combined with bitewing radiography, are still most common techniques for examining the marginal integrity of restorations [40]. Visual changes adjacent to restorations such as discolorations, staining, or dentinal shading may be caused by a lot of factors: only one of them being secondary caries lesions [9]. In this study, the two dentists using ICDAS II for visual assessment could not detect if there was caries beneath the restoration margin.

Fluorescence based caries detection devices may have challenges in detecting caries around amalgam margins. A number of studies have concluded that measuring fluorescence is not suitable for detecting caries around restoration margins or beneath dental sealants due to false positive readings [17, 41-43]. The CR Clinicians’ Report (March 2012) found that existing restorations interfered with readings [44]. Furthermore, fluorescence based technologies do not give any information about lesion size or depth, and the light does not penetrate beneath the tooth surface due to the scattering of light from stain, plaque, organic deposits and surface features such as pits and fissures [45, 46]. SPECTRA images were not able to discern the restoration margins (Figs. **2** & **3**). The amalgam had very strong fluorescence which interfered with the ability of the device to detect the margins of the restoration or measure the enamel adjacent to the amalgam.

DIAGNODent is also a fluorescence-based device but uses a different wavelength from SPECTRA, (660 nm) and is a point measurement so it was able to pick up some information from the tooth structure adjacent to the amalgam with some interference from the material [47]. As measurements were taken further from the amalgam margin DIAGNODent sensitivity and specificity did improve but was still hampered by the fluorescence from the amalgam. The fluorescent nature of the amalgam itself contributed to the elevated DIAGNODent numbers and therefore lower specificity for sound samples at the margin. Overall DIAGNODent was less consistently able to differentiate between sound and carious samples.

The Canary System is able to measure an area of 1.5 mm. in diameter and up to 5 mm below the tooth surface. It provides a Canary Number (ranging from 0 -100) from an algorithm combining the PTR and LUM readings, which are directly linked to the status of the enamel or root surface crystal structure [48] (Fig. **2**). A Canary Number of less than 20 indicates healthy crystal structure. A Canary Number greater than 70 indicates a large lesion that may justify restoration. Canary Numbers falling between 20 and 70 indicate the presence of early carious lesions or cracks that may require restoration [49], particularly at restorative margins [50]. If the caries is located beneath a healthy layer of enamel, the Canary measures both healthy tissue and caries in the area of the beam. The healthy crystal structure overlying the caries dampens the signal, decreasing the Canary Number but still keeping it above the healthy range. An *in-vitro* study has shown that The Canary System can detect caries beneath an intact opaque fissure sealant, more accurately than visual examination or DIAGNODent [51] demonstrating that the sealant may dampen but not eliminate the signal from the caries lesion. In this *in-vitro* study, The Canary System was able to examine the margins of the amalgam restoration and up to 2 mm. beyond the amalgam margin and discern if there was healthy or carious tissue present. There have been some published case reports on caries detection around the margins of amalgam restorations [52-54] with The Canary System. This *in-vitro* study helps to validate their findings.

## CONCLUSION

Investigators using SPECTRA and visual examination using ICDAS II rankings were unable to detect caries around the margins of the amalgam restoration *in vitro*. The Canary System and DIAGNOdent were able to differentiate between sound and carious tissue at the margin of the restoration. DIAGNOdent results had larger variation, less reliability, and poorer accuracy. Using this *in-vitro* model, The Canary System has the potential to detect secondary caries around amalgam restorations more accurately than the other investigated modalities. The Canary System may be able to provide a clinician with another device to examine the margins of amalgam restorations.

## Figures and Tables

**Fig. (1):**
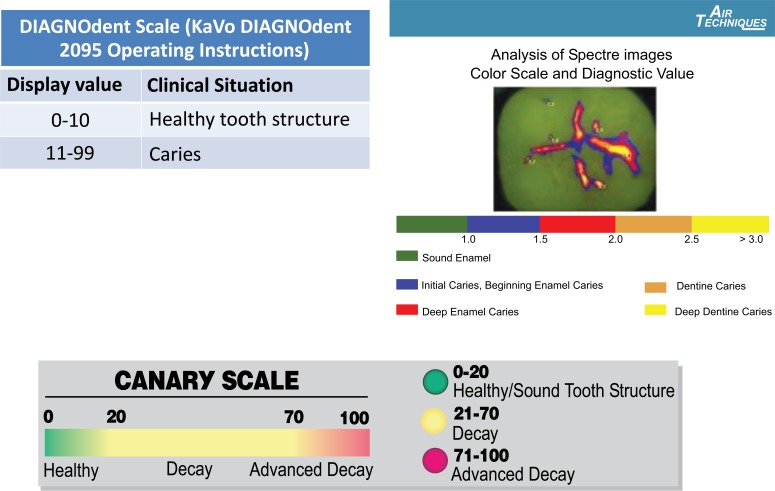
Scales for the caries detection devices employed in this study. A) The DIAGNOdent Scale developed by Lussi et al. (2001) for detection of occlusal caries lesions. B) The Canary Scale. The Canary Scale is a relative scale of 0 - 100 that reflects the state of tooth mineralization and crystallization. This is a graduated scale where lower numbers indicate sound enamel and higher numbers indicate more advanced tooth decay. SPECTRA.

**Fig. (4):**
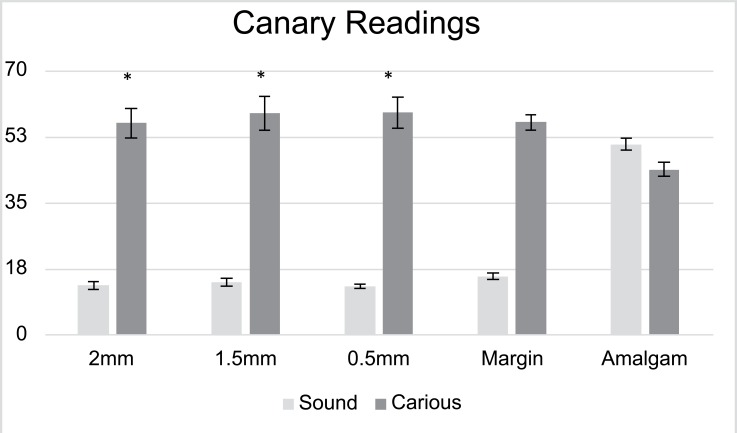
Mean Canary Numbers at MOR, 0.5, 1.5 and 2 mm from the margin into tooth structure for sound teeth and carious teeth. Asterisks (*) indicate statistical significance at *P*<0.05.

**Fig. (5):**
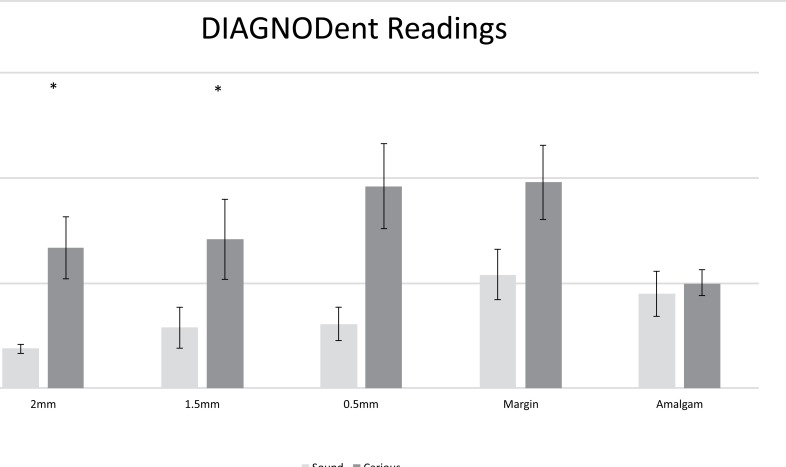
Mean peak values for DIAGNOdent at MOR, 0.5, 1.5 and 2 mm from the margin into tooth structure for sound teeth and carious teeth. The asterisks indicate statistical significance at *P*<0.05.

**Table 1:** Sensitivity and Specificity for Canary System & Diagnodent.

	Sensitivity			Specificity	
Carious Teeth	CN	DD	Sound Teeth	CN	DD
2 mm	95%	52%	2 mm	100%	100%
1.5 mm	95%	45%	1.5 mm	92%	85%
0.5 mm	96%	65%	0.5 mm	100%	85%
MOR	100%	74%	MOR	85%	54%

**Table 2:** Two Sample T-Test for Canary System & Diagnodent.

	T-Test (Equal Variances)			T-Test (Equal Variances)	
Carious Teeth	CN	DD	Sound Teeth	CN	DD
2 mm	0.005	0.282	2 mm	<<0.001	0.020
1.5 mm	0.002	0.292	1.5 mm	<<0.001	0.255
0.5 mm	0.001	0.032	0.5 mm	<<0.001	0.268
MOR	<<0.001	0.013	MOR	<<0.001	0.560
